# Rates of Serious Intracellular Infections in Autoimmune Disease Patients Receiving Initial Glucocorticoid Therapy

**DOI:** 10.1371/journal.pone.0078699

**Published:** 2013-11-19

**Authors:** Kiyoshi Migita, Toru Arai, Naoki Ishizuka, Yuka Jiuchi, Yasuharu Sasaki, Yasumori Izumi, Tetsuyuki Kiyokawa, Eiichi Suematsu, Tomoya Miyamura, Hiroshi Tsutani, Yojiro Kawabe, Ryutaro Matsumura, Shunsuke Mori, Shiro Ohshima, Shigeru Yoshizawa, Kenji Kawakami, Yasuo Suenaga, Hideo Nishimura, Toyohiko Sugimoto, Hiroaki Iwase, Hideyuki Sawada, Haruhiro Yamashita, Shigeyuki Kuratsu, Fumitaka Ogushi, Masaharu Kawabata, Toshihiro Matsui, Hiroshi Furukawa, Seiji Bito, Shigeto Tohma

**Affiliations:** Japanese National Hospital Organization (NHO)-EBM study group for Adverse Effects of Corticosteroid Therapy (J-NHOSAC), Meguro, Tokyo, Japan; National Taiwan University Hospital, Taiwan

## Abstract

**Background/Aims:**

The Japanese National Hospital Organization evidence-based medicine (EBM) Study group for Adverse effects of Corticosteroid therapy (J-NHOSAC) is a Japanese hospital-based cohort study investigating the safety of the initial use of glucocorticoids (GCs) in patients with newly diagnosed autoimmune diseases. Using the J-NHOSAC registry, the purpose of this observational study is to analyse the rates, characteristics and associated risk factors of intracellular infections in patients with newly diagnosed autoimmune diseases who were initially treated with GCs.

**Methodology/Principal Findings:**

A total 604 patients with newly diagnosed autoimmune diseases treated with GCs were enrolled in this registry between April 2007 and March 2009. Cox proportional-hazards regression was used to determine independent risk factors for serious intracellular infections with covariates including sex, age, co-morbidity, laboratory data, use of immunosuppressants and dose of GCs. Survival was analysed according to the Kaplan-Meier method and was assessed by the log-rank test. There were 127 serious infections, including 43 intracellular infections, during 1105.8 patient-years of follow-up. The 43 serious intracellular infections resulted in 8 deaths. After adjustment for covariates, diabetes (Odds ratio [OR]: 2.5, 95% confidence interval [95% CI] 1.1–5.9), lymphocytopenia (≦1000/μl, OR: 2.5, 95% CI 1.2–5.2) and use of high-dose (≧30 mg/day) GCs (OR: 2.4, 95% CI 1.1–5.3) increased the risk of intracellular infections. Survival curves showed lower intracellular infection-free survival rate in patients with diabetes, lymphocytopaenia and high-dose GCs treatments.

**Conclusions/Significance:**

Patients with newly diagnosed autoimmune diseases were at high risk of developing intracellular infection during initial treatment with GCs. Our findings provide background data on the risk of intracellular infections of patients with autoimmune diseases. Clinicians showed remain vigilant for intracellular infections in patients with autoimmune diseases who are treated with GCs.

## Introduction

Despite the considerable benefits of glucocorticoids (GCs) in controlling serious inflammation and improving the functional status of a plethora of disorders [1], serious adverse effects dampen the enthusiasm for their use, particularly long-term [2]. Bacterial and fungal infections are the most common serious infections occurring in patients receiving GCs [3], although intracellular infections are also a concern [4]. Moderate- to high-dose GC therapy leads to an increased risk of opportunistic infections, including intracellular infections [5], [6]. However, there is little information regarding the rate of intracellular infection in a large series of patients receiving GCs in clinical practice. Most studies of GCs toxicity are retrospective and risk factors for intracellular infections have not been completely elucidated in prospective studies. Because immunosuppression induced by GCs is broad, a wide variety of pathogens can infect hosts treated with GCs [7]. Thus, the clinician must anticipate both usual and unusual infections, including intracellular infections. There is a paucity of well-controlled studies detailing the infectious risks, particularly concerning intracellular infections, and much of the information is in the form of case reports and literature reviews [8], [9]. In addition, many of these reports offer little detail regarding the dose or duration of GC administration.

The Japanese National Hospital Organization evidence-based medicine (EBM) Study group for Adverse effects of Corticosteroid therapy (J-NHOSAC) is a Japanese non-interventional prospective study, based on a nationwide registry of severe adverse events (AEs) in newly diagnosed autoimmune disease patients treated with moderate doses of GCs in a clinical setting [10]. This original strategy can be used to determine the exact incidence of serious infections and their risk factors and using the J-NHOSAC registry we previously reported that infections were the most common AEs occurring in newly diagnosed autoimmune disease patients, who were initially treated with GCs [10]. The aim of this study was to describe cases of intracellular infections, and to identify risk factors for their occurrence.

## Materials and Methods

### Patients and Study design

Patients were eligible for the study if they were initially treated with GCs for a recently diagnosed (within 4 weeks prior to study entry) autoimmune disease using established criteria. The cohort start date was defined as the time of initiation of the first GC prescription. The autoimmune diseases registered in this study were as follows. Rheumatic diseases: SLE (systemic lupus erythematosus), MCTD (mixed connective tissue disease), polymyositis, dermatomyositis, vasculitis, Behçet disease, systemic scleroderma, AOSD (adult onset Still disease), Sjögren syndrome, rheumatoid arthritis, autoimmune bullous diseases, and anaphylactoid purpura. Neurological diseases: multiple sclerosis, myasthenia gravis, and CIDP (chronic inflammatory demyelinating polyradiculoneuropathy). Gastro-hepatobiliary diseases: ulcerative colitis, autoimmune hepatitis, autoimmune pancreatitis, and PBC (primary biliary cirrhosis). Interstitial lung diseases: idiopathic interstitial pneumonia and collagen vascular disease preceded by interstitial pneumonia. Primary glomerular diseases: rapidly progressive glomerulonephritis, chronic glomerulonephritis, and nephrotic syndrome.

A total of 604 patients with newly diagnosed autoimmune disease were enrolled between April 1, 2006 and March 31, 2008, and regularly followed concerning the occurrence of GC-related adverse effects. The observation period ended on March 31 2009. Before implementation of this study, institutional review board and ethics committee approvals of the protocol and the consent to participate in the study and consent to publish were obtained from each of the participating patients. The study was approved by the ethical committees of the National Hospital Organization (NHO) central internal review board (No. 0512014, 2006). Written informed consent was obtained from each individual.

### Data collection

Data from all participating physicians were entered into the J-NHOSAC database at the data centre of the International Medical Center of Japan in Tokyo, Japan via the HOSPnet internet system. Serious infections were defined as those that led to hospitalization or death or required intravenous antibiotic treatment. If a serious infection was identified from the patient's medical records provided by participating doctors. If not already provided, additional information regarding all serious infections was requested, including causative organism.

### Data on study entry

The past comorbid condition of each patient was reviewed by each of the principle physicians. These conditions included renal, neurological, endocrine, cardiovascular and pulmonary diseases as well as cancer and stroke. In addition, the incidences of specific conditions, including pre-existing pulmonary tuberculosis, hepatitis viral infection (HBV, HCV), diabetes, hyperlipidaemia, arrhythmia and performance status (Karnofsky score) were assessed. The physicians also provided information on smoking or drinking habits and history of tuberculosis (TB). At entry, patients underwent chest X-ray and were screened for surface hepatitis B virus antigen (HBs Ag) and anti-HCV antibodies.

### Outcome variables

At the start of the study, standardized lists were used to document AEs, which were classified using the System Organ Class (SOC) of the Medical Dictionary for Regulatory Activities (MedDRA; version 11.1). All physicians documented episodes of infection requiring medical care and death certificates and the causes of deaths that occurred during the follow-up periods. Serious infections occurring during the observation periods were counted. Serious infections (≥ grade 3, by common terminology of Adverse Events v3.0 [CTCAE]), were defined as life-threatening, requiring hospitalization and/or intravenous antibiotic therapy, or leading to significant disability/incapacity1 [11]. Infections were coded by anatomic site and by causal organism. Cytomegalovirus (CMV) infection was defined as CMV end-organ disease, in which the signs and symptoms of affected organs (pneumonia, gastrointestinal disease, or hepatitis) plus CMV antigenaemia were present. The diagnosis of CMV antigenaemia was defined by a positive CMV PP65 antigenaemia assay [12]. A diagnosis of Pneumocystis pneumonia (PCP) was made according to the established criteria for PCP [13]. The diagnosis of PCP was considered presumptive if a patient fulfilled the clinical and radiographic conditions in the absence of evidence of other infectious diseases and in the presence of either a positive PCR test for *Pneumocystis jirovecii* DNA (qualitative PCR analysis by SRL, Tokyo, Japan) or increased serum β-D-glucan levels above the upper limit of normal (ULN) (Fungitec G test MK; Seikagaku, Tokyo, Japan) and responded to standard treatments for PCP with trimethoprim/sulfamethoxazole (TMP/SMX) or pentamidine isethionate. Both the PCR test for *P. jirovecii* DNA and the serum BDG test are commercially available, validated, and officially approved as clinical laboratory tests by the Ministry of Health, Labour, and Welfare in Japan.

### Follow-up data

Patients were followed up every 3 months by the chief physician for each of the NHO hospitals, who collected clinical findings (disease activity, severity, performance status, blood pressure, body weight) and laboratory data (complete blood cell count, biochemistry, and urinalysis). The telephone interview concerning the health assessment and the presence of GCs-related AEs was conducted against few patients who were moved or transferred to another hospital at the end of cohort. However, overall outcome was not available from 14 patients (2.3%) at the end of study. In statistical analysis, we excluded these participants without final outcome data.

### Medications

Details of GCs, immunosuppressants, and biologics were recorded at each visit, including the route of administration and dose. We categorized GC exposure according to the mean daily dose throughout the follow-up period for each patient. We calculated “dose equivalents” of prednisolone as follows: 1 mg of prednisolone  = 5 mg of cortisone  = 4 mg of hydrocortisone  = 1 mg of prednisone  = 0.8 mg of triamcinolone  = 0.8 mg of methylprednisolone  = 0.15 mg of dexamethasone  = 0.15 mg of betamethasone [14].

### Statistical analysis

We identified the risk factors of intracellular infections by univariate and multivariate Cox-proportional hazard models analysis The variables included in the analysis were: age, sex, types of primary autoimmune diseases, comorbidities (diabetes, renal diseases, cardiovascular diseases and interstitial lung diseases), medications (average dose of GC, the use of immunosuppressive agents) and performance status (Karnofsky score) or laboratory data on entry (serum albumin, serum IgG, lymphocyte counts). Factors included in the Cox-proportional multivariate hazard model were those associated with the status (case or control) of univariate analysis with a significance level of *p*<0.05. The receiver operating characteristics (ROC) curve was used to identify the best threshold value for continuous variables that were predictors of the occurrence intracellular infections. Both univariate and multivariate analyses were performed, using the occurrence of at least one serious infection during the follow-up period as the outcome. Results are expressed as odds ratios (ORs) with 95% confidence intervals (95% CIs). Qualitative variables were compared using the chi-square test (or Fisher's exact test when appropriate), and quantitative variables were compared using the Mann-Whitney *U*-test. Two-sided *p* values less than 0.05 were considered statistically significant. The analysis was conducted using SAS (version 9.1, SAS Institute, Cary, NC, USA).

## Results

### Baseline characteristics

The baseline characteristics are shown in Table 1. A total 604 patients with newly diagnosed autoimmune diseases initially treated with GCs from 51 hospitals were enrolled in the J-NHOSAC registry between April 2006 and March 2008. The analysis was performed on all patients with a mean follow-up period of 1.9±0.64 years, for a total of 1105.8 patient-years. There were 358 (59.3%) females and 246 (40.7%) males, with a mean age of 59.5±16.8 (range 21 to 82) years. All patients received GCs at entry and the mean GCs dose for the first month was 50.4±63.1 mg/day. Concomitant immunosuppressive therapies are summarized in Table 1. Two-hundred and eighty-three patients (46.9%) were treated with immunosuppressants or biologics. The mean dose (lengths) of the immunosuppressive agents as follow; tacrolimus (n = 24); 1.7±0.71 mg/day (376.7±277.7 days), cyclosporine (n = 76); 123.4±39.6 mg/day (409.3±285.6 days), methotrexate (n = 87), 5.7±2.5 mg/week (524.4±368.6 days), oral cyclophosphamide (n = 22); 55.4 mg ±10.9 mg/day (244.1±288.7 days), intravenous cyclophosphamide (n = 17); 577.1±201.6 mg (2.6±1.9 times), etanercept (n = 12) 124.7 mg ±72.0 mg/month (328.8.1±261.6 days), infliximab (n = 7) 248.2 mg ±134.5 mg/month (226.3.±187.3 days), adalimumab (n = 3) 87.7 mg ±2.2 mg/month (288.1±53.6 days). Thirty four patients had experienced previous tuberculosis infection. Prophylaxis was performed in 69 patients using isoniazid (INH) and in 138 patients using TMP/SMX.

**Table 1:** Baseline characteristics of the 604 patients with autoimmune diseases treated with glucocorticoid.

			n = 604(%)
Females			358(59.3)
Mean age (years)			59.5±16.8
BW (kg)			55.9±10.9
Karnofsky score			79.4±18.3
Primary disease			
	Rheumatic disease		313(51.8)
		SLE	38(6.3)
		MCTD	10(1.7)
		Polymyositis	18(3.0)
		Drmatomyositis	16(2.6)
		Vasculitis	46(7.6)
		Behçet's disease	5(0.8)
		Systemic sclerisis	12(2.0)
		AOSD	13(2.2)
		Sjögren's syndrome	6(1.0)
		Rheumatoid arthritis	136(22.5)
		Autoimmune bullous diseases	7(1.2)
		Anaphylactoid purpura	6(1.0)
	Neurological disease		25(4.1)
		Multiple sclerosis	4(0.7)
		Myasthenia gravis	20(3.3)
		CIDP	1(0.2)
	Gastro-Hepatobiliary disease		79(13.1)
		Ulcerative colitis	20(3.3)
		Autoimmune hepatitis	51(8.4)
		Autoimmune pancreatitis	4(0.7)
		PBC	4(0.7)
	Interstitial lung disease		133(22.0)
	Primary Glomerular disease		54(8.9)
		Rapidly progressive glomerulonephritis	7(1.2)
		Chronic glomerulonephritis	18(3.0)
		Nephrotic syndrome	29(4.8)
Previous TB			34(5.6)
Co-morbidity			
	Cardiovascular disease		
		CVA	20(3.3)
		Ischemic heart disease	25(4.1)
		Hypertension	128(21.2)
		Arrythmia	19(3.1)
		Others	12(2.0)
	Metabolic disease		
		Hyperlipidemia	149(24.7)
		Hyperuricemia	44(7.3)
		Diabetes	65(10.8)
		CKD	32(5.3)
Antimicrobial prophyraxis			
	Isoniazid		69(11.4)
	Trimethoprim-sulfamethoxazole		138(22.8)

Abbreviations: AOSD;Adult Onset Still's Disease, CIDP;Chronic inflammatory demyelinating polyneuropathy, CDK;Chronic kidney disease, CVA;Cerebro-vascular accident, SLE;Systemic lupus erythematosus, MCTD;Mixed connective-tissue disease, PBC;Primary biliary cirrhosis, TB;Tuberculosis Data are expressed as mean ± standard deviation or number (percentage).

### Incidence of intracellular infections

**Figure 1:**
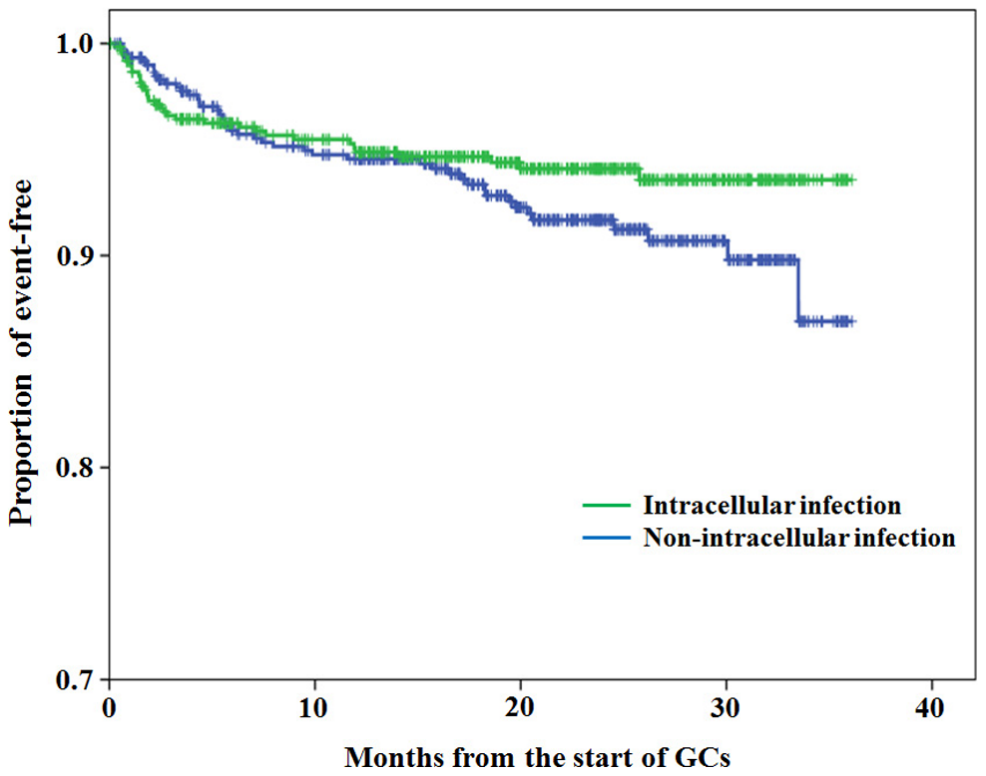
Kaplan-Meier curves for time to first intracellular infections and non-intracellular infections according to time since initiation of GC treatment.

During the overall follow up periods, 127 serious infections that required hospitalization and/or intravenous antibiotics and/or resulted in death, occurred in 71 patients. The incidence rate of serious infections was 114.8 events/1,000 person-years. These serious infections (n = 127) included 43 intracellular infections. Pathogens identified in 33 patients with intracellular infections are listed in Table 2 and included CMV (n = 14), herpes zoster (n = 7), Epstein–Barr virus (n = 1), *P. jirovecii* infection (n = 7), *Mycobacterium tuberculosis* (n = 2) non-tuberculosis mycobacterium (NTM, n = 1) and *Listeria monocytogenes* (n = 1). The site of these intracellular infections is listed in Table 2. A multi-intracellular infection was reported in eight patients. The intracellular infections occurred earlier and faster than normal infections and 50% of intracellular infections occurred within 4 months of starting GCs. In contrast, non-intracellular serious infections occurred gradually compared with serious intracellular infections (Figure 1).

**Table 2:** Details of 31 patients with intracellular infections.

					Co-morbidity		Immunosuppressive agents				
Patient No.	Infection	Site of infection	Patient age/sex	Primary disease	Cardiovascular disease	Metabolic disease	Immunosuppressant	Biologics	Months from the start of Steroid	Multi-Intracellular infection	Fatal outcome
**1**	CMV	Viremia	73/M	SLE	Ischemic heart diseas	Diabetes			1		
**2**	CMV	Viremia	62/M	Interstitial pulmonia		Hyperlipidemia			1		
						Diabetes					
**3**	CMV	Viremia	58/M	Autoimmune hepatitis			Azathioprine		1		
**4**	CMV	Viremia	82/F	Systemic sclerisis					2		**+**
**5**	CMV	Viremia	79/F	Vasculitis	Hypertension		Tacrolimus		1	**+**	
**6**	CMV	Viremia	42/F	Polymyositis			Cyclosporin		1	**+**	
**7**	CMV	Viremia	40/F	Drmatomyositis			Cyclosporin		1		
**8**	CMV	Lung	76/M	Vasculitis	CVA	Diabetes	Azathioprine		3	**+**	
**9**	CMV	Lung	71/M	Interstitial pulmonia		Hyperlipidemia	Cyclophosphamide		2	**+**	**+**
**10**	CMV	Lung	70/F	Rheumatoid arthritis		Hyperlipidemia	Cyclosporin		12	**+**	**+**
**11**	CMV	Liver	81/M	Vasculitis	CVA	Diabetes			2	**+**	**+**
					Ischemic heart diseas						
**12**	CMV	Liver	51/F	SLE		Hyperlipidemia			1		
						Hyperuricemia					
**13**	CMV	Colon	21/M	Ulcerative colitis					5		
**14**	CMV	Colon	85/F	Ulcerative colitis	CVA				2		
					Hypertension						
**15**	PCP	Lung	72/M	Interstitial pulmonia	Ischemic heart diseas		Cyclophosphamide		3		
					Hypertension						
**16**	PCP	Lung	71/M	Rheumatoid arthritis			Methotrexate	Etanercept	8		
							Tacrolimus				
**17**	PCP	Lung	66/M	Rheumatoid arthritis	Hypertension	Hyperlipidemia	Methotrexate		2		
						Diabetes					
**18**	PCP	Lung	62/M	Interstitial pulmonia		Diabetes	Cyclophosphamide		3	**+**	**+**
							Cyclosporin				
**19**	PCP	Lung	61/M	Interstitial pulmonia					2		
**20**	PCP	Lung	81/F	Vasculitis	Hypertension	Hyperuricemia			2		
						Diabetes					
**21**	PCP	Lung	68/F	Interstitial pulmonia	Hypertension	Hyperuricemia	Cyclosporin		19		**+**
**22**	Herpes zoster	Skin	74/M	Rheumatoid arthritis	Hypertension	Diabetes	Tacrolimus		2	**+**	**+**
**23**	Herpes zoster	Skin	58/M	Interstitial pulmonia	Hypertension		Cyclophosphamide		12		
							Cyclosporin				
**24**	Herpes zoster	Skin	39/M	Myasthenia gravis					26		
**25**	Herpes zoster	Skin	24/M	MCTD		Hyperuricemia			7		
**26**	Herpes zoster	Skin	77/F	Autoimmune hepatitis	Hypertension				14		
**27**	Herpes zoster	Skin	72/F	Interstitial pulmonia	Hypertension	Hyperlipidemia			12		
**28**	Herpes zoster	Skin	53/F	Interstitial pulmonia	Arrythmia				1		
**29**	TB	Lung	91/M	Autoimmune bullous diseases	CVA				3		
					Ischemic heart diseas						
					Hypertension						
					Arrythmia						
**30**	TB	Lung	68/F	Interstitial pulmonia		Hyperlipidemia			9		
**31**	NTM	Lung	70/M	Rheumatoid arthritis					20		
**32**	EBV	Liver	58/F	Vasculitis	Hypertension	Hyperlipidemia			1		
**33**	Listeria	Brain	73/M	Vasculitis	Hypertension	Hyperuricemia			6		**+**
		(Meningitis)			Arrythmia						

Abbreviations: CMV;Cytomegalovirus, PCP; Pneumocystis jiroveci pneumonia, TB; Tuberculosis, NTM; Nontuberculous mycobacterious, EBV; Epstein-Barr virus, SLE; Systemic lupus erythematosus, MCTD; Mixed connective-tissue disease, CVA; Cerebrovascular accident.

### Risk factors for intracellular infections

**Figure 2:**
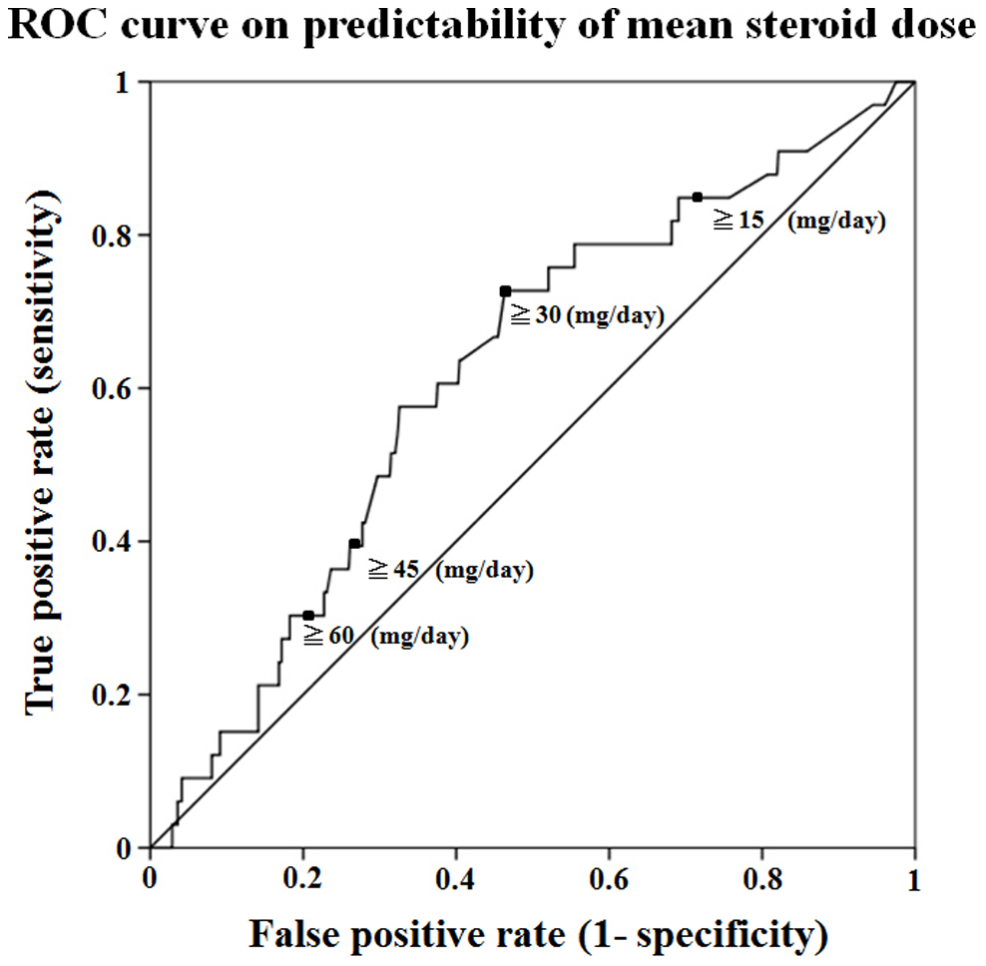
ROC curve on predictability of mean steroid dose. Receiver operating characteristics (CRC) curve for different cut-off values, as shown in the graph, of mean steroid dose to predict intracellular infections.

The case-control study involved 33 patients with serious intracellular infections and 559 patients without serious intracellular infection. To identify differences in risk factors that contributed to the development of intracellular or non-intracellular infections, we compared baseline data between patients with serious intracellular infections and those with non-intracellular serious infections. The positive predictive value (PPV) of mean steroid dose to identify intracellular infections was shown in the receiver operating characteristics (ROC) curve (Figure 2). Cut-off value of steroid dose was made at 30 mg/day based on this ROC curve analysis and this value was entered into the Cox hazard model. Univariate Cox regression analysis showed a significant increased risk of serious intracellular infections with male gender, the presence of diabetes, use of high-dose (≧30 mg/day) of GCs, lower Karnofsky score and lymphocytopenia (≤1000/μl). No significant association was found for the use of immunosuppressants or biologics as risk factors. In the multivariate Cox regression analysis, high-dose (≧30 mg/day) of GCs (OR = 2.4 [95% CI, 1.1–5.3], the presence of diabetes (OR = 2.5 [95% CI, 1.1–5.9]) and lymphocytopenia (<1000/μl, OR = 2.5 [95% CI, 1.2–5.2]) were significantly associated with an increased risk for serious intracellular infections (Table 3).

**Table 3:** Predictors of serious intracellular infection in the COX-hazard model.

Predictors	Intracellular infection	No intracellular infection	Univariate analysis		Multivariate Analysis	
	n = 33	n = 559	OR (95%CI)	*p* value	OR (95%CI)	*p* value
Age	64.5±16.5	59.4±16.7	1.255 (0.994–1.585)	0.056		
			[10-year increment]			
Gender Male	19(57.6%)	219(39.2%)	2.164 (1.084–4.318)	0.029	1.843 (0.873–3.891)	0.109
Female	14(42.4%)	340(60.8%)				
Primary disease						
Rheumatic disease	18(54.5%)	287(51.3%)	1.082 (0.545–2.147)	0.822		
Neurological disease	1(3.0%)	24(4.3%)	0.657 (0.090–4.808)	0.679		
Gastro-Hepatobiliary disease	4(12.1%)	74(13.2%)	0.891 (0.313–2.535)	0.829		
Interstitial pulmonia	10(30.3%)	122(21.8%)	1.730 (0.822–3.637)	0.149		
Primary Glomerular disease	0	52(9.3%)				
Diabetes	8(24.2%)	57(10.2%)	2.923 (1.316–6.489)	0.008	2.548 (1.106–5.872)	0.028
Previous TB	1(3.0%)	33(5.9%)	0.512 (0.070–3.744)	0.509		
Treatment						
Dose of prednisolone (mg/day)	61.0±55.6	49.1±62.5				
≧30.0mg/day	24(72.7%)	269(48.1%)	3.043 (1.141–6.550)	0.004	2.417 (1.100–5.308)	0.028
Immunosuppressant	14(42.4%)	207(37.0%)	1.300 (0.652–2.594)	0.456		
Biologics	1(3.0%)	17(3.0%)	0.985 (0.135–7.212)	0.988		
Karnofsky score	73.9±18.5	79.8±18.3	0.836 (0.718–0.975)	0.022	0.901 (0.757–1.072)	0.239
			[10 score increment]			
Laboratory data						
Serum creatinine (mg/dl)	0.76±0.33	0.78±0.64	0.999 (0.535–1.865)	0.998		
Serum albumin (mg/dl)	3.30±0.71	3.45±0.76	0.737 (0.471–1.155)	0.183		
Serum IgG (mg/dl)	1811.5±733.8	1855.8±866.1	1.000 (0.999–1.000)	0.894		
WBC (/μl)	7637.2±3551.5	7769.3±3917.9	1.000 (1.000–1.000)	0.909		
Lymphocyte count (/μl)	1239.6±631.2	1554.5±714.5				
≦1000	12(38.7%)	108(20.8%)	2.347 (1.139–4.835)	0.021	2.517 (1.207–5.248)	0.014

Abbreviations: OR; Odds ratio, 95% CI; 95% confidence interval Data are expressed as number (percentage) or mean ± SD.The hazard ratios for serious intracellular infection were estimated using the Cox proportional hazard model.Patients (n = 12) without final outcome data were excluded in this analysis.

### Survival curves

**Figure 3:**
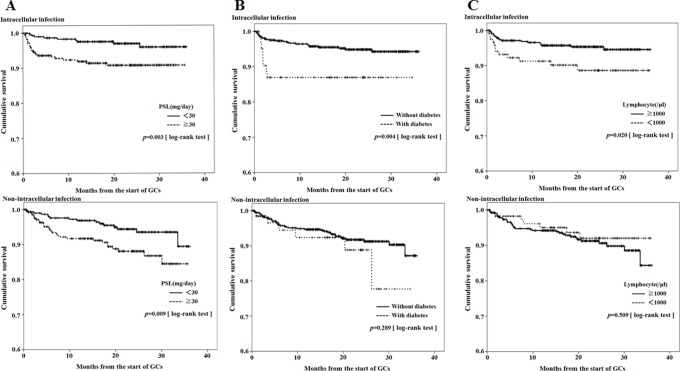
Infection (intracellular or non-intracellular) -free survival curves. Figure 3A: Cumulative probability of intracellular or non-intracellular infection-free survival for the complete follow-up period between patients receiving high-dose of GC (≥30 mg/day) and those not receiving high-dose of GC. 3B: Cumulative probability of intracellular or non-intracellular infection-free survival for the complete follow-up period in patients with or without diabetes at time of presentation. 3C: Cumulative probability of intracellular or non-intracellular infection-free survival for the complete follow-up period in patients with or without lymphocytopenia (<1000/μl) at time of presentation.

Patients with serious intracellular infections were found to have significant associations to diabetes or low lymphocyte counts compared with those with non-intracellular serious infections. Therefore, Kaplan-Meier survival curves were plotted for the occurrence of the first serious intracellular or non-intracellular -infections stratified by the presence of high dose GCs (≧30 mg/day), (Figure 3A), diabetes (Figure 3B) and lymphocytopenia (<1000/μl, Figure 3C). Reduced survival time to the occurrence of first intracellular infections was significant in patients receiving high dose GCs, diabetes or lymphocytopenia. In contrast, reduced survival time to the occurrence of first non-intracellular serious infections was significant only in patients receiving high dose GCs, but not in patients with diabetes or lymphocytopenia.

## Discussion

GCs are still widely used in the treatment of autoimmune diseases and are thought to be associated with increased risk of infection as well as other well-established adverse effects [15]. However, the extent to which GC contributes to the increased risk of intracellular infections is not completely understood. Despite the fact that intracellular infections rank high amongst common causes of death in immunological diseases [16], they have not been studied specifically in autoimmune diseases. In the current study, we showed that the overall rate of intracellular infections is high in patients with newly diagnosed autoimmune diseases treated with GCs.

Our study showed that intracellular infections occurred more frequently and quickly (most within 4 months from the start of GCs) among patients with autoimmune diseases and some intracellular infections contributed to morbidity and mortality. A high frequency of opportunistic infections complicating autoimmune diseases has been reported previously [17]. The augmented risk is due in part to the underlying immune dysfunction associated with these disorders [18], but also due to immunosuppressive therapies used to treat these disorders. It is often difficult to separate intracellular infections because of underlying diseases (which may be immunosuppressive) and infections related to GC-induced immunosuppression. However, the type of primary autoimmune disease was not identified as a risk factor for intracellular infection. In contrast, we demonstrated that the presence of diabetes, lymphocytopaenia and the initial use of high-dose GCs (≧30 mg/day), elderly age and male gender were independent predictors for serious intracellular infections in patients with newly diagnosed autoimmune diseases.

To date, there have been few studies evaluating the exposure of GCs in newly diagnosed autoimmune diseases and reporting the rate of intracellular infection. A number of published papers have evaluated the risk of infections in connective tissue diseases [5], [19], [20], where the exposure of GC, outcomes and risk measurements were not consistent across the studies. Methodological differences between previous studies and ours are that previous investigators used retrospective analysis or hospitalization record techniques to ascertain infection occurrence. In our study, we reviewed each patient's complete medical records and obtained objective confirmatory evidence of infection including microbiologic cultures and radiographic imaging prospectively. Use of these detailed medical records enabled us to obtain information regarding a broad range of pathogens and infection sites.

The striking incidence of intracellular infections seen in our study suggests the presence of cell-mediated immune deficiency in patients with autoimmune diseases receiving moderate-doses of GCs [21]. From epidemiologic studies, treatment with a daily dose of less than 5 mg of prednisolone or equivalent slightly increased risk of infection [22], whereas daily doses between 20 to 40 mg caused a marked increased risk [23]. However, the true incidence of intracellular infections or their risk factors have not been adequately demonstrated in a prospective study. One of the objectives of this study was to identify possible baseline risk factors for the development of serious intracellular infections in autoimmune disease patients treated with moderate-doses of GCs (>30 mg/day, mean of first month). Previous studies identified high-doses of GCs, lymphocytopaenia, use of immunosuppressants, low IgG levels and diabetes as independent variables associated with increased risk of intracellular infection [6], [16], [24]–[27]. Although the differences between these studies and our results are significant (design, disease evaluated, doses of GCs and variables analysed), it is noteworthy that both studies identified lymphocytopaenia, high doses of GCs and diabetes as independent variables associated with increased risk of intracellular infections [6], [26]–[28]. However, we found no association between concomitant use of immunosuppressive agents or biologics and an increased risk of intracellular infections. How GCs alter the host immune system and influence the development of infection is incompletely understood. Human host immunity against intracellular pathogens is dependent on effective cell-mediated immune responses [29]. GCs can impair phagocyte functions and suppress cell-mediated immunity, possibly increasing the risk of intracellular infections [1].

In general, diabetes is associated with an increased susceptibility to infection [30]. However, no adequate data exists for the effects of diabetes on risk of intracellular infection. Our study clearly demonstrated that the presence of diabetes is a risk factor for intracellular infection in patients treated with GCs. This may be due to abnormalities of the host immune response, particularity defects of neutrophils, macrophages, chemotaxis, adhesion and intracellular killing that are attributed to hyperglycaemia and may explain reduced phagocytosis [31]. Disorders of cell-mediated immunity are often associated with CMV and PCP. CMV is the most common opportunistic pathogen detected in organ transplant recipients and can cause a variety of clinical symptoms in patients with disordered cell-mediated immunity including pneumonitis colitis, hepatitis and retinitis [9]. An increased risk of PCP in association with lymphocytopaenia has been similarly reported in patients with connective tissue diseases [32], [33]. Clinicians should maintain a high degree of suspicion for the development of unusual intracellular infections in patients receiving moderate doses of GCs. Initiation of GC therapy requires careful pre-treatment evaluation. Screening for latent tuberculosis, diabetes, cellular-immune dysfunction (lymphocytopaenia) and consideration of prophylaxis should be considered [21], [34]. Evaluation and management of suspected intracellular infections in patients receiving newly administered GC treatment must include aggressive diagnostic approaches and empiric treatment.

Our study has a number of limitations that should be considered. We analysed only variables related to cellular or humoral immune monitoring, without considering other aspects that have demonstrated influence on the incidence of intracellular infections. The determination of laboratory or clinical findings was performed exclusively at baseline, which may hinder analysis of steroid therapy modulation of patient immune status. Furthermore, we did not perform a comparison with patients not treated with GCs. Therefore, we could not compare patients treated with GCs or other immunosuppressive agents alone. The nature of the study design did not allow us to exclude completely the role of potential confounders, such as disease severity, whereby patients with more-severe disease (and thus at high risk of infection) are more likely to receive steroids, and thus is a major concern. However, this potential bias was unavoidable in our observational study. Future well-designed prospective studies are required to confirm our findings.

In conclusion, substantial numbers of intracellular infections occurred in patients with newly diagnosed autoimmune diseases, who were treated with GCs. We showed that patient baseline demographics, such as concomitant diabetes, lymphocytopaenia, and initial high-dose GCs (>30 mg/day) might predict an increased incidence of intracellular infections. Our findings suggest strategies for efficient pre-treatment risk screening, intensive surveillance, prophylaxis and pre-emptive therapy for intracellular infections in patients with newly diagnosed autoimmune diseases, which may eventually lead to the improvement of prognosis and survival.
